# Understanding Physiology in the Continuum: Integration of Information from Multiple -*Omics* Levels

**DOI:** 10.3389/fphar.2017.00091

**Published:** 2017-02-27

**Authors:** Kubra Kamisoglu, Alison Acevedo, Richard R. Almon, Susette Coyle, Siobhan Corbett, Debra C. Dubois, Tung T. Nguyen, William J. Jusko, Ioannis P. Androulakis

**Affiliations:** ^1^Department of Pharmaceutical Sciences, School of Pharmacy and Pharmaceutical Sciences, University at Buffalo, BuffaloNY, USA; ^2^Department of Biomedical Engineering, Rutgers University, PiscatawayNJ, USA; ^3^Department of Biological Sciences, University at Buffalo, BuffaloNY, USA; ^4^Department of Surgery, Rutgers Robert Wood Johnson Medical School, New BrunswickNJ, USA; ^5^BioMaPS Institute for Quantitative Biology, Rutgers University, PiscatawayNJ, USA; ^6^Department of Chemical Engineering, Rutgers University, PiscatawayNJ, USA

**Keywords:** transcriptomics, proteomics, metabolomics, pharmacogenomics, inflammation, glucocorticoids, LPS

## Abstract

In this paper, we discuss approaches for integrating biological information reflecting diverse physiologic levels. In particular, we explore statistical and model-based methods for integrating transcriptomic, proteomic and metabolomics data. Our case studies reflect responses to a systemic inflammatory stimulus and in response to an anti-inflammatory treatment. Our paper serves partly as a review of existing methods and partly as a means to demonstrate, using case studies related to human endotoxemia and response to methylprednisolone (MPL) treatment, how specific questions may require specific methods, thus emphasizing the non-uniqueness of the approaches. Finally, we explore novel ways for integrating *-omics* information with PKPD models, toward the development of more integrated pharmacology models.

## Introduction

**FIGURE 1:**
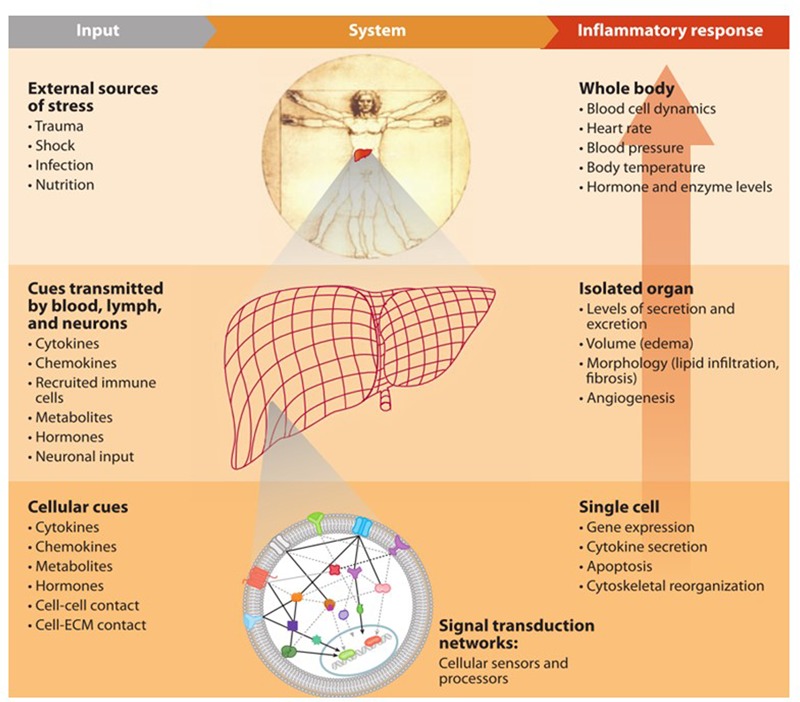
**Multiscale nature of inflammatory response**.

Life is complex at all scales. From a single cell to the whole body, there are myriad intricate mechanisms that control every aspect of this complexity, **Figure 1**. The ultimate aim of biomedical sciences is to establish a thorough understanding of how these control mechanisms function when in a healthy state and how the control is lost (or shifted to a new mode) when symptoms of a disease are displayed, in order to explain the observed phenotypic changes with the known paradigms at the molecular level. Our ability to collect information about molecular events in our bodies has tremendously increased with great advancements in technology. However, we still have a long way to go finding the best ways to fully utilize this information.

Numerous -*omics* tools are available, each of which makes it possible to observe the physiologic responses at their complementary level. They enable the examination of a broad array of cellular or systemic elements and functions through the use of vast amounts of quantitative or semi-quantitative data from various levels of biological organizations (Richards et al., 2010). Systems biology rises on these new technologies and its most current and significant challenge is developing methods to integrate the vast amount of information into a conceptual framework that is holistic, quantitative and predictive (Kritikou et al., 2006). The intent is to reach a thorough understanding of the biological mechanisms driving different processes in our bodies and ascertain insights how to manipulate these processes for our benefit.

**FIGURE 2:**
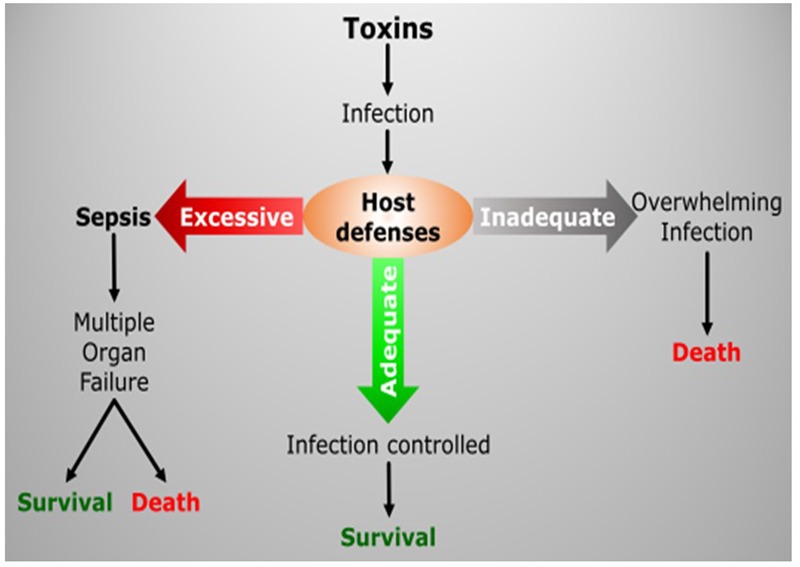
**Inflammatory response is a complex process, which has to be tightly regulated in order to balance the defense mechanisms of the host with the severity of infection/tissue damage.** Loss of this control in favor of either side may have fatal consequences.

Inflammation is one of those core processes through which we produce responses against various stressors, such as pathogens or trauma. It is a complex and multiscale biological phenomenon that needs to be orchestrated under tight regulation (Laroux, 2004). Factors inducing physiological stress are sensed and translated into biological cues that transmit signals throughout organs and down to the cellular level. These incoming signals are then recognized and processed to produce a response in a dynamic and highly regulated manner. Collective cell responses change the dynamic of biological metrics, manifesting change at the individual organ level and ultimately throughout the entire body. Under normal conditions, the outcome of inflammation is the mounting of required immune response for pathogen elimination or regeneration following injury; however, in any instance of dysregulation of this complex process, the uncontrolled response can induce further damage or lead to a non-sustainable systemic disease state (Bone, 1996), **Figure 2**. Considering its critical role in our survival, inherent complexity and intricate relationships with other essential physiologic processes; inflammation and inflammatory diseases are among the research fields that can benefit from adapting the systems approach. In this respect, the emerging -*omics* tools are very promising, since they offer the advantage of observing the inflammatory response at a much broader level together with the ability to analyze multiple variables simultaneously which empowers the application of systems analysis for rationalizing and modeling the course of physiologic events.

Comprehending the continuum of physiologic responses to pathologic stimuli is essential for making sense of how molecular changes develop to induce observable symptoms of a particular disease. Drugs, i.e., pharmacologic stimuli, are intended to reverse this disease progression and reduce the symptoms. For most cases, physiologic effects of drugs are also complex and include re-directing physiologic responses to alleviate symptoms of a particular pathologic condition as well as inducing adverse-effects associated with off-target reactions. Analyses of the physiologic effects of drugs by monitoring a handful of markers for the targeted effects has been used for building models of drug action for many years. Extensive -*omics* analyses done at multiple physiologic levels, however, also impacted this research area tremendously and initiated a shift from classic pharmacokinetic/pharmacodynamic modeling (PK/PD) toward a systems approach defined as quantitative systems pharmacology (QSP) (Iyengar et al., 2012; Jusko, 2013; Androulakis, 2015, 2016). The ultimate direction for this field is the realization of personalized and precision medicine by building progressively more accurate drug action models. However, the first steps toward these goals involve devising methods to fully utilize the wealth of information produced by the extensive analyses of pharmacologic responses.

This paper is centered on integrating information from multiple physiologic levels. We focused on how critical relationships are shaped over time during the development of the response to a systemic inflammatory stimulus and in response to an anti-inflammatory treatment. The systems approach allowed us to track the continuum of physiologic responses through their evolution and in relation to multiple dynamics running in harmony. We extracted the coherent dynamic responses represented in the -*omics* analyses at multiple physiologic levels and integrated them using multiple approaches. The analyses described in the sections that follow include metabolic and transcriptional responses to endotoxemia, an experimental model in humans that recapitulates the dynamics of systemic inflammatory response. We then switch to an anti-inflammatory therapy and focus on the effects of a commonly used synthetic glucocorticoid in liver. This analysis represents a more direct integration approach, in which we evaluate the concordance of the hepatic response to the drug treatment at gene and protein expression levels. Finally, we discuss a network-based approach that integrates -*omics* information with existing pharmacokinetic/pharmacodynamic models.

## Integration of -*Omics* Data: Why and How?

“Why” and “how” are, likely, the most critical questions that need to be addressed as we aggressively enter the -*omics* era. The answer to “why” is straightforward: life is a complex organization of functional entities each manifesting the actions and activities of appropriate levels through corresponding markers at a suitable level, broadly described as *-omics* data. Therefore, the *-omics* data provide a snapshot of the system across multiple levels of organization. The suffix *“ome”* is used to identify groups of objects sharing common characteristics, either descriptive or functional. At an elementary level we routinely think of genomic (sequence of the genome), transcriptomic (expression of the genome), proteomic (expression of the proteome), metabolomics (expression of the metabolome) and the list is continuously expanded to include the epigenome (Koch, 2016), interactome (Karagoz et al., 2016), regulome (Cheng et al., 2015), microbiome (Althani et al., 2016) to name only a few.

Clearly, one of the key challenges in the *-omics* era is “integration” and most precisely *how* to integrate. It is important to realize that although the various *-omics* components at some elementary level augment the number of descriptors, the augmentation is not passive, i.e., it is not simply increasing the dimensionality of the space. Rather, it introduces additional layers of knowledge which are not independent of other layers, that is the various *-omics* data are functionally related, most likely implicitly among themselves. Seemingly simple, yet profound, challenges emerge (Gomez-Cabrero et al., 2014) while a wide range of methods have been proposed and extensively reviewed (Rogers, 2011; Meng et al., 2014). Broadly speaking we will argue that integration can be accomplished either (a) via statistical means considering the computational challenges associated with simultaneous analysis of disparate data sources (Meng et al., 2014; Gligorijevic et al., 2016) or (b) realizing that the *-omics* information comes together, eventually, in the form of a yet to be determined, dynamics model expressing interactions, cross-functionality and constraints (Chen et al., 1999; Palsson, 2002; Joyce and Palsson, 2006; Hyduke et al., 2013). However, the purpose of the present discussion is not to provide a detailed account of the field and/or the approaches, but rather articulate, through the use of specific examples: (1) how the nature of the data can guide, and to some extent constrain, the type of question that can be asked; and (2) how questions can guide the approach that need to be developed and pursued. Both of these topics will be discussed in the context of specific applications related to inflammation and anti-inflammatory drugs. Each of the sections below is primarily concerned with addressing one particular questions and outlining one, of the likely many approaches that exist for addressing it.

## Data-Driven Integration of Transcriptomic and Proteomic Data at the Same Level of Physiological Organization

The first case study refers to a scenario where the *-omics* data reflect different processes, principally within the same cell type, reflecting a sequence of events. Likely the most characteristic example of this would be transcriptomic and proteomic information. The fundamental question arising here is how to upgrade the longitudinal information quantifying gene and protein expression simultaneously, for a particular cell type in response to an external perturbation or environmental condition. In a simplistic way, one can view this problem as an extension of the so-called *central dogma*. We demonstrate the alternatives by focusing on the analysis of transcriptomic and proteomic data obtained from the *in vivo* response of a rodent model following bolus administration of synthetic glucocorticoids. Studies focusing on understanding the relationship between global mRNA transcription and protein translation have produced mixed results, many of which concluded that the transcriptomic and proteomic data is far from being easily described as complementary (Greenbaum et al., 2003; Hegde et al., 2003; Nicholson et al., 2004; Waters et al., 2006; Haider and Pal, 2013; Motta and Pappalardo, 2013; Castiglione et al., 2014). Nevertheless, both data types reflect the dynamics of the cellular response to any given perturbation, thus capture critical information reflecting different facets of the response. Without considering any type of functional relation between transcriptomic and proteomic data, each can be considered independently or *in tandem*. In any “data driven” query, the approach undertaken largely reflects the “biases” imposed by the type of question one wishes to address (Androulakis et al., 2007).

In the sections that follow, we will address a basic question: assuming that the response to drug elicits changes at both the transcriptional and translational level, is it reasonable to assume that one would focus on gene transcripts and corresponding protein abundance *both* exhibiting substantial temporal changes relative to base? (Note: in the analysis that follows, we assume that the base line expression levels, i.e., in the absence of the drug, are represented by mRNA and protein abundance levels prior to drug administration). In a number of previous publications, we have illustrated the analysis of longitudinal data with a time-varying base line (Androulakis et al., 2007; Yang et al., 2007b, 2008a, 2009b, 2012a,b; Yang E.H. et al., 2009; Almon et al., 2008a,b; Nguyen et al., 2009, 2010a,b, 2011, 2014a; Ovacik et al., 2010; Scheff et al., 2010a; Swiss et al., 2011). However, temporal relations among time-varying quantities are known to be non-trivial, extending far beyond the classic view of correlation (Qian et al., 2001).

### Synthetic Glucocorticoids

Synthetic corticosteroids (CS), such as MPL, are widely used anti-inflammatory and immunosuppressive agents for the treatment of many inflammatory and auto-immune conditions including organ transplantation, rheumatoid arthritis, lupus erythematosus, asthma and allergic rhinitis (Swartz and Dluhy, 1978; Barnes, 1998). The mechanism of action of CS drugs is basically magnifying the physiological actions of the endogenous glucocorticoid hormones, which have anti-inflammatory properties depending on their secretion level and the time at which they are secreted. These hormones also have diverse effects on a variety of physiological processes including carbohydrate, lipid and protein metabolism, immune-regulation, bone homeostasis and developmental processes (Barnes, 1998; Vegiopoulos and Herzig, 2007). The well-established molecular mechanism of action for CS includes the passive diffusion of the highly lipophilic CS molecule through the cell membrane and binding to the cytosolic glucocorticoid receptor, which is held inactive through the association with heat shock proteins (Schaaf and Cidlowski, 2002). Binding of the drug to the receptor causes conformational changes, phosphorylation and activation of receptor, resulting in the formation of a homodimer of the drug receptor complex (Schaaf and Cidlowski, 2002; Oakley and Cidlowski, 2011). This activated complex translocates into the nucleus and binds to regulator sites, glucocorticoid regulatory elements (GREs) in the DNA, resulting in the regulation of transcription rate. In addition to direct binding, the activated complex can regulate gene expression by other mechanisms including tethering and composite binding to other transcription factors, activators, or repressors (Barnes, 1998; Schaaf and Cidlowski, 2002). Studies have shown that CS can regulate pathways by signaling through receptors in a transcription-independent manner, although the exact mechanisms for the non-genomic effects are still unclear (Schaaf and Cidlowski, 2002). While short-term use of CSs is beneficial for reducing the inflammation, long-term use is associated with serious side-effects – including hyperglycemia, negative nitrogen balance, and fat redistribution, leading to complications including diabetes, muscle wasting, osteoporosis, hypertension, cataracts, peptic ulcers, and reduced resistance to infection and adrenal insufficiency following withdrawal of therapy (Andrews and Walker, 1999; Morand and Leech, 1999). The undesirable metabolic effects of CS cannot usually be separated from their favorable anti-inflammatory effects since most actions manifest via the same glucocorticoid system.

**FIGURE 3:**
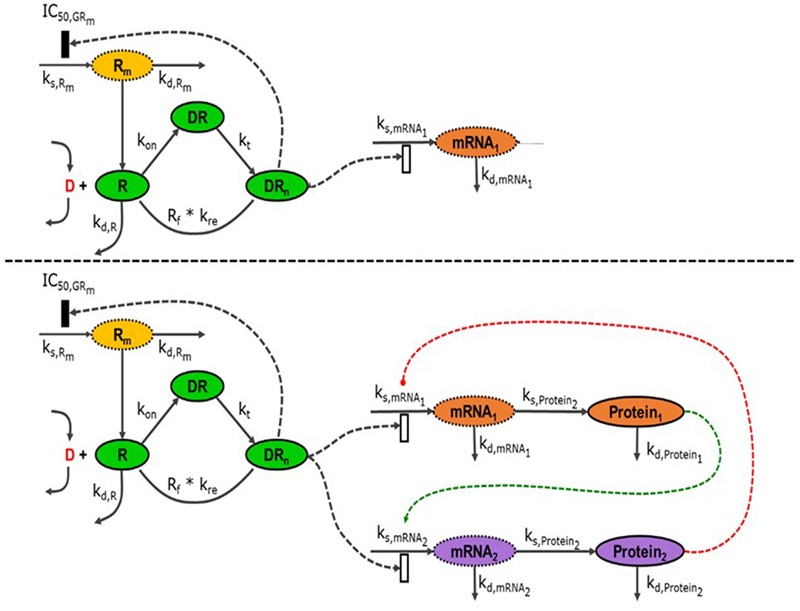
**(Top)** Schematic representation of the network-based indirect response model to MPL (D). Once the drug-receptor complex translocates into the nucleus (DR_n_), it induces its effects on the transcription of its target genes (mRNA_1_, mRNA_2_) either by stimulation (open rectangle) or by inhibiting it (closed rectangle). **(Bottom)** When transcribed messages are translated to the active proteins (Protein_1_, Protein_2_), they can also have effects, either stimulatory (green) or inhibitory (red), on the transcription of target genes affected by MPL. All these effects are considered as indirect as there might be additional biological processes in between.

The analysis of the diverse physiological effects of synthetic glucocorticoids has been the subject of many pharmacokinetic and pharmacodynamic modeling efforts. A series of models has been developed to explain the dynamics of receptor regulation and enzyme induction following MPL administration (Sun et al., 1998; Ramakrishnan et al., 2002), **Figure 3** (top). The models were progressively enhanced to capture the effects of the drug under several doses and dosing regimens. However, these models were based on the data generated by traditional message quantification methods that only allow measurements of single end points. Because of the diverse effects of CS and different molecular mechanisms potentially involved in these actions, a high-throughput transcriptomic, i.e., microarray, approach was effective in gaining better understanding of the temporal and tissue-specific effects of CS on different pathways and functions (Almon et al., 2007a,b). The diversity of the available models describing gene induction by MPL have been expanded to several pharmacogenomics models that explain the response of numerous genes with various dynamic patterns (Almon et al., 2003, 2005, 2007a,b; Jin et al., 2003; Nguyen et al., 2014a). Our earlier studies in collaboration with Prof. Jusko, characterized global dynamics of the systems that are regulated by CS at the transcriptional level across multiple tissues in adrenalectomized (ADX) and intact male rats, following single and chronic dosing of MPL enabling us to: (a) develop transcription-level understanding of MPL (acute vs. chronic) effects; (b) elaborate on tissue-specific transcriptional differences; (c) assess MPL-induced dose- and tissue-specific transcriptional regulation; and (d) assess circadian dynamics and regulation of intact and MPL-dosed animals (Yang et al., 2007b, 2008a; Yang E.H. et al., 2009; Almon et al., 2008a; Nguyen et al., 2010a,b, 2014a; Ovacik et al., 2010; Scheff et al., 2010a, 2011).

Although transcriptional information is useful and highly relevant, direct profiling of the protein expression changes and integration of the information from proteomic data will provide deeper insights into CS actions (Nouri-Nigjeh et al., 2014). Recently, high-throughput, ion current-based liquid chromatography/mass spectrometry (LC/MS), allowed comprehensive and accurate profiling of the tissue proteome (Tu et al., 2012). Using this methodology, the temporal changes in the expression of almost a 1000 proteins in rat liver following MPL administration were characterized (Nouri-Nigjeh et al., 2014). The analysis of the combined transcriptomic and proteomic data confirmed that significant indirect regulation by MPL was evident, notably indicated by significant changes in mRNA and protein levels in the absence of glucocorticoid responsive promoter elements. With this new information from the protein expression level, it was possible to evaluate complementarities between transcription and translation of the target genes and elaborate on the interplay between gene and protein expression in liver toward a more complete understanding of the indirect mechanisms of action of MPL (Kamisoglu et al., 2014). This data confirmed that MPL effects are propagated across a network of interacting genes and proteins, **Figure 3** (bottom). Without considering any type of functional relation between transcriptomic and proteomic data, each can be considered independently (Androulakis et al., 2007) and using statistical methods one can analyze temporal trends at each level. This was the approach undertaken recently in our lab and an extensive discussion of the methods and results was recently presented (Kamisoglu et al., 2014).

### Animal Model, Proteomic and Transcriptomic Data

#### Proteomic Studies

Sixty adrenalectomized (ADX) Wistar rats were injected with 50 mg/kg methylprednisolone (MPL) intramuscularly and sacrificed at 12 different time points between 0.5 and 66 h post-dosing (five animals/time point). Five animals, injected with saline and sacrificed at random time points in the same time window, served as controls. In order to remove the high concentrations of blood protein, it was necessary to use perfused tissue for proteomic analyses, which precluded the use of the same tissues employed for transcriptomics (below). Proteins from perfused and flash frozen livers were extracted, digested and analyzed using a nano-LC/LTQ/Orbitrap instrument. The Nano Flow Ultra-high Pressure LC system (nano-UPLC) consisted of a Spark Endurance autosampler (Emmen, Holland) and an ultra-high pressure Eksigent (Dublin, CA, USA) Nano-2D Ultra capillary/nano-LC system, with a LTQ Orbitrap mass spectrometer (Thermo Fisher Scientific, San Jose, CA, USA) used for detection. Protein quantification was based on the area under the curve (AUC) of the ion-current-peaks. A more extensive description of the experimental setup and the analytical methodology can be found in the previously published study (Nouri-Nigjeh et al., 2014).

#### Transcriptomic Studies

Forty-three ADX Wistar rats were given a bolus dose of 50 mg/kg MPL intravenously. Animals were sacrificed at 16 different time points between 0.25 and 72 h post-dosing. Four untreated animals sacrificed at 0 h served as controls. The mRNA expression profiles of the liver were arrayed via Affymetrix GeneChips Rat Genome U34A (Affymetrix, Inc.), which contained 8800 full-length sequences and approximately 1000 expressed sequence tag clusters (Jin et al., 2003). This dataset was previously submitted to the GEO (GSE490). We have previously presented extensive analyses and studies of the transcription response to acute and chronic administration of MPL on multiple tissues (Almon et al., 2008a,b; Yang et al., 2008a; Nguyen et al., 2010a, 2014a; Ovacik et al., 2010).

All animal experiments were performed at the University of Buffalo and protocols adhered to “Principles of Laboratory Animal Care” (NIH publication 85-23, revised in 1985) and were approved by the University at Buffalo IACUC committee.

### Integration and Analysis of Transcriptomic/Proteomic Data

Assessment of the problem greatly reflects the approach(es) taken (Androulakis et al., 2007) whereas in (Kamisoglu et al., 2015b) we described multiple approaches to integrate this temporal information from the proteome level with the corresponding dynamics in the transcriptomic level through data-driven approaches. In the analysis that follows we addressed the question in two different ways. First, we identified transcripts and proteins which are over-expressed post MPL administration. The intersection of the two sets indicates genes from which both transcription and translation is significantly altered. The *simultaneous* consideration enables us to consider the following question: what is the dynamic correlation of the subset of MPL-regulated transcripts and proteins? As mentioned earlier, and shown later, the relation is far from trivial. However, such analysis, only depicts part of the picture. It is highly likely that transcriptional events do not manifest themselves at the protein levels and also post-transcriptional alterations may not include transcriptional alterations. For such an analysis, the two -*omics* data need to be separated and analyses performed. Therefore, in the former case the mining of the data is concurrent but the functional interpretation separate, whereas in the latter the mining of the data is separate but the functional analysis concurrent. The case study discussed in the following section demonstrates both approaches. Once again, it is important to realize that we only discuss two of the many questions which could be posed and addressed and the presentation is by no means exhaustive or all-encompassing.

#### Augmenting the Feature Vector: Clustering of Concatenated Dataset

This analysis will help us identify the genes which were differentially expressed both at the transcriptional and translational levels. Data analysis for both proteomic and transcriptomic datasets started first by filtering for differential expression over time. Proteins and transcripts with differential temporal profiles were determined by using EDGE (Leek et al., 2006). We employed within-class differential expression to extract profiles that have a differential expression over time (Storey et al., 2005, 2007; Leek et al., 2006). Integration of these two datasets for any further analysis required matching the object identifiers which was achieved through running a comparison between two filtered datasets in Ingenuity Pathway Analysis (IPA, Ingenuity^®^ Systems)^1^.

**FIGURE 4:**
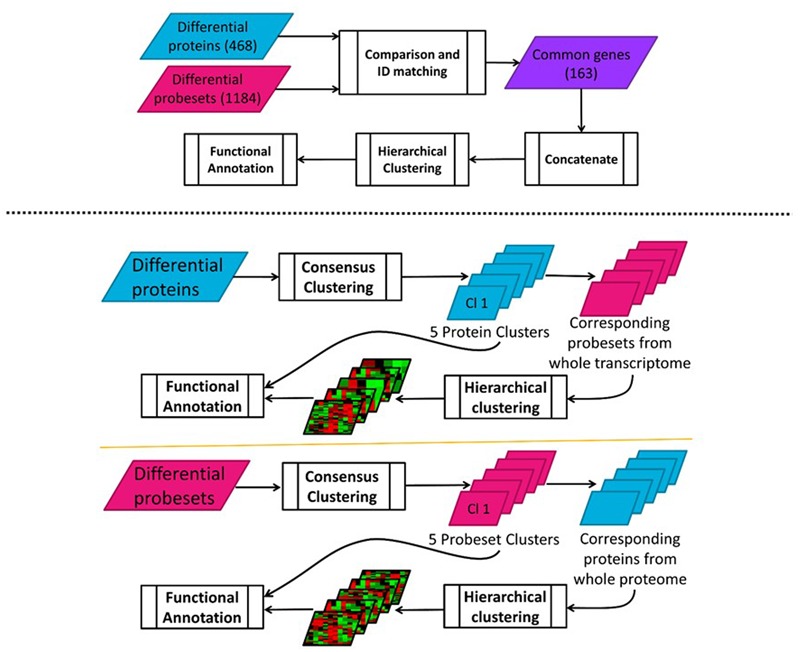
**(Top)** Clustering of concatenated transcriptomic-proteomic MPL data workflow; **(Bottom)** Sequential transcriptomic and proteomic – forward and reverse – clustering analysis of MPL data workflow.

In order to find potential co-regulatory relationships at these two levels, hierarchical clustering was used for first-pass analysis. For this purpose, temporal transcriptomic and proteomic data for the common genes were first concatenated and then clustered using the clustergram function in the Bioinformatics toolbox of MATLAB (Mathworks, Natick, MA, USA). The two clusters obtained by using correlation as the distance metric. The workflow is illustrated in **Figure 4** (top).

This study aimed to compare and contrast the transcriptional and translational changes in liver induced by the exposure to a synthetic CS at a pharmacological dose. Although high-throughput -*omics* analyses have been obtained from samples collected from two independent studies; the strain of experimental animals, dose and type of pharmacologic agent, sampled tissue, sampling procedures, and most of the time points for sample collection were the same for these studies. These conditions allowed us to assume that the experiments are similar enough to conduct individual and integrated bioinformatics analyses.

The preprocessing before performing the first-pass analysis involved identifying the significant genes whose both transcripts and proteins existed in the individual datasets. Differential expression analysis through EDGE identified that 475 out of 959 proteins and 1624 out of around 8800 transcripts had temporal profiles that significantly varied over time (meeting *p*-value < 0.05 and *q*-value < 0.01 cut-offs). After this filtering step, both datasets were fed into IPA in order to match distinct identifiers used (Swiss-Prot IDs for proteins and Affymetrix IDs for transcripts). A comparison between two datasets indicated that 163 genes were commonly found in both transcriptomic and proteomic datasets; i.e., both mRNAs and proteins corresponding to these genes were differentially expressed over time.

The analysis is described in greater in detail (Kamisoglu et al., 2015b). However, we briefly report here that this analysis identified two dominant patterns: one with corresponding mRNA and protein expression profiles essentially parallel in direction, and another where the directionality was reversed. The early response appears to be the most critical time period during which mRNA and protein expression profiles change direction. Genes in Cluster 1 display up-regulation for both mRNA and protein expression profiles in the first 8 h, followed by down-regulation, most markedly in the transcriptional profiles. In the second cluster of response profiles, down-regulation dominates for transcriptional profiles; however, corresponding protein expression profiles are not parallel. While down-regulation is observed in these transcripts most notably in the first 8 h; expression of the same proteins seems to be up-regulated in the same time frame. After the 8th hour; both transcriptional and protein expression profiles approach basal levels, though from opposite directions; elevated mRNA levels start to be down-regulated and reduced protein levels start to be up-regulated. Functional annotation of the genes in these two clusters, indicated that the first cluster contains a number of genes coding for heat shock proteins, which take part in the negative regulation of CS signaling through direct protein-protein interaction with glucocorticoid receptor to prevent its translocation to nucleus (Chrousos and Kino, 2005). Complementary transcriptional and proteomic profiles of these genes indicated that this is a negative feedback control induced by MPL delivery, which is regulated at the transcriptional level. Proteins functioning in the regulation of protein degradation and translation machinery were also among the genes in the first cluster, implying that these processes are also controlled at the transcriptional level after CS exposure. In contrast, functions enriched by the genes in the second cluster appear to be regulated at post-transcriptional levels, likely through control of mRNA processing, initiation of protein translation or protein stability, since the transcriptional profiles are not emulated by protein expression, (Waters et al., 2006). Among these functions, most notable are the modulation of oxidative stress, lipid metabolism and bile acid biosynthesis. Concurrent analysis of promoter region identified upstream regulators that can explain the observed changes in gene/protein expression based on the prior knowledge of expected effects between the upstream regulators and target genes/proteins in the dataset.

#### Two-Way Sequential Clustering of Individual Proteomic and Transcriptomic Datasets

While the hierarchical clustering analysis described above identifies the potential co-regulatory schemes for the genes in the intersection of transcriptomic and proteomic datasets; it fails to capture the dynamics in the rest of the genes which may also show differences in expression over time, although they may not co-exist in both datasets. In order to evaluate the overall dynamic patterns and extract the most useful information integrating these two datasets, a consensus clustering (Nguyen et al., 2009) method was applied to these two datasets separately. First, proteins with differential temporal profiles were clustered using *p*-values of 0.05 for significant clusters and an agreement level of 0.70 for the genes in each cluster. Then, probe sets corresponding to the proteins in each cluster were identified through the comparison function in IPA as before. Temporal profiles of these probe sets corresponding to the proteins were compiled and separately sub-clustered through the less stringent hierarchical clustering method, again using clustergram function in MATLAB. The reverse of the same procedure was also performed – starting from transcriptional analysis and continuing with the corresponding proteomic analysis. Here, differential transcriptional profiles were first determined and then clustered using the same procedures described above. As with the previous analysis, proteins that correspond to the probe sets within each of these clusters were then identified and sub-clustered. The workflow is illustrated in **Figure 4** (bottom). Functional annotations of proteins and transcripts at each level of analysis were conducted in IPA by running a core analysis for each cluster and evaluating the enriched canonical pathways (at *p*-value threshold of 0.05) and predicted upstream regulators obtained in IPA.

Summarizing the observations described in great detail in (Kamisoglu et al., 2015b) we note that EDGE identified 475 out of 959 while the ensuing consensus clustering revealed five coherent temporal profiles containing 217 of the 475 regulated proteins. Of the 217 clustered proteins, 158 showed regulation of at the mRNA level as well. This analysis was repeated in the reverse direction; i.e., starting from the transcriptomic dataset and progressing to the proteomic dataset. 1624 of the probe sets were differentially expressed, 1132 of those were in five clusters obtained by consensus clustering. Only 217 of these 1132 probe sets had corresponding proteins in the proteomic dataset. Compared to the first part of sequential clustering analysis, considerably fewer proteins actually correlate with the transcriptional profile of their respective clusters. Considering that protein expression is a more reliable predictor of function, the annotation analysis was based on the proteomic data in this part of the analysis. Elaboration on the results enabled a more complete characterization of the functional implications and relations among gene and proteins (Kamisoglu et al., 2015b).

## Data-Driven Integration -*Omics* Data Across Multiple Levels of Physiological Organization

The second study addresses a question of increasing importance. Blood sampling is the most widely utilized way of probing dynamic responses, and it has tremendous translational potential as it is the most readily accessible sample in humans. Metabolomics in particular is becoming a readily available tool given the ability of the serum metabolome to capture biological responses at a higher level (Minami et al., 2009; Fitzpatrick and Young, 2013; Kosmides et al., 2013; Kaddurah-Daouk et al., 2014; Kell and Goodacre, 2014; Carroll et al., 2016). Likely one of the most celebrated examples was the Inflammation and the Host Response to Injury, so-called, “*Glue Grant*,” aiming at providing a blue-print of the host response to injury and trauma based on sophisticated analysis of blood samples (Nathens et al., 2005; Cuenca et al., 2011; Tompkins, 2015). However, despite the fact that the majority of studies attempting to integrate various types of -*omics* information, analyses based on blood measurement add an extra level of complexity. Namely, circulating metabolites originate from a wide variety of tissues and organs and eventually accumulate in systemic circulation. Therefore, although the various data structures provide actual information describing the host response, the fact that the origin of each type of information is not unique, as well as non-specific, significantly complicates the analysis. One such example, to be discussed shortly, relates to combining transcriptomic and metabolomics information from blood samples. In this direction, the question we pursued was based on the hypothesis that the drastic changes in the immediate environment of blood leukocytes might have an adaptive effect on shaping their transcriptional response in the regulation of metabolism in conjunction with the initial inflammatory stimuli. The coupling of leukocyte transcriptomic and systemic metabolomics information may enable us to provide a more complete picture of the drivers of the response of immune cells under inflammatory conditions.

### Human Endotoxemia

Elective administration of bacterial endotoxin (lipopolysaccharide; LPS) to healthy human subjects has been used as a reproducible experimental procedure providing mechanistic insights into how cells, tissues and organs respond to systemic inflammation. Low doses of LPS transiently alter many physiologic and metabolic processes in a qualitatively similar manner to those observed after acute injury and systemic inflammation (Lowry, 2005; Calvano and Coyle, 2012), thus allowing the analysis of the responses to infectious stress at multiple physiologic levels. This model has been extensively employed for the development and assessment of rational clinical therapies to prevent or attenuate systemic inflammatory response syndrome (SIRS) (Calvano and Coyle, 2012).

Response to endotoxemia is closely associated with alterations in metabolism. Inflammatory processes change the direction of the substrate flow from the periphery toward splanchnic organs while also triggering the release of catabolic signals in order to meet increased energy and substrate demands (Fong et al., 1990; Khovidhunkit et al., 2004); and hence, considerably alter the levels of plasma metabolites. Individual changes in the major metabolites, such as some lipids, amino acids, and glucose, are previously documented for the case of human endotoxemia (Fong et al., 1990). However, an untargeted bioinformatics-empowered approach to elucidate the effects of endotoxemia on plasma metabolite levels is lacking.

Analysis of the complete metabolic response to systemic inflammation is of special interest since metabolic composition of a tissue is uniquely altered in response to stimuli due to collective effects of the regulations at various levels of cellular processes including transcription, translation and signal transduction. Concentrations of metabolites in a sample at a given time, i.e., the “metabolome” (Nicholson and Lindon, 2008), can be thought of as the metabolic fingerprint representative of the state of body at that time and provide information on the dominant regulatory mechanisms. The emerging field of metabonomics, combines this unique metabolic information with bioinformatics approaches to provide an integrated temporal picture of the interactions in the system (Nicholson, 2006; Holmes et al., 2008). Since the metabolic phenotype is determined by eventual production of metabolites through the complex cellular processes trickling down from transcription, translation and signal transduction, this field offers promise in advancing the knowledge in many clinical conditions. For endotoxemia, understanding the alterations in plasma metabolome is critical; since, metabolite levels impacts the regulation of anti-inflammatory defenses, in turn, through steering critical cellular processes in immune cells (Pearce and Pearce, 2013).

Global transcriptomic studies of circulating leukocytes in experimental human endotoxemia previously elucidated the intricate regulatory schemes governing the inflammatory response (Calvano et al., 2005; Nguyen et al., 2011). However, inflammatory response is also closely associated with alterations in metabolism. In Kamisoglu et al. (2013) we discussed the drastic effect of a mild inflammatory stimulus on the homeostasis of the whole-body metabolism. This single level analysis uncovered the temporal patterns in the host metabolism reflecting collective impacts of regulations at various organs and at multiple levels of cellular processes including transcription, translation and signal transduction. For endotoxemia, understanding the alterations in plasma metabolome is critical, since metabolite levels impact the regulation of anti-inflammatory defenses, in turn, by directing critical cellular processes in immune cells (Pearce and Pearce, 2013).

Building on this knowledge; we integrated the transcriptional response of leukocytes with systemic metabolic response to understand how inflammation-induced changes in the composition of plasma, in turn, affecting the transcriptional processes in the leukocytes.

#### Metabolomic Studies

Archived blood plasma samples, which had been flash frozen, were used in this proof-of-principle study. These samples were collected from 19 healthy subjects, ages 18–40. Fifteen of the subjects (11 males and 4 females; mean age of 22.7) were administered National Institutes of Health (NIH) Clinical Center Reference Endotoxin, at a bolus dose of 2 ng/kg body weight as previously described Alvarez et al. (2007); Jan et al. (2009) and Jan et al. (2010). Four control subjects (three males and one female; mean age of 22.2) were administered placebo (saline). During the protocol, subjects received a solution of 5% dextrose and 0.45% saline crystalloid. Blood draws were conducted sequentially at *t* = 1, 2, 6, and 24 h from both groups, samples were inventoried and stored at -80°C until the analysis. Metabolomic analysis was performed by Metabolon (Durham, NC, USA) according to previously published methods (Evans et al., 2009). The resulting extracts were subjected to either liquid chromatography (LC) or gas chromatography (GC) followed by mass spectroscopy (MS) analysis. Identification of known chemical entities was based on comparison to metabolomic library entries of purified standards. Complete details of the profiling of plasma metabolome are previously described (Kamisoglu et al., 2013, 2015a).

#### Transcriptomic Studies

For the transcriptomic study, four subjects (one female and three male) received LPS at a bolus dose of 2 ng/kg body weight and four subjects (one female and three male) received saline. Blood samples were collected before (*t* = 0 h) and 2, 4, 6, 9, and 24 h after LPS administration. Leukocytes were recovered by centrifugation; total cellular RNA was isolated from the leukocyte pellets and hybridized onto Hu133A and Hu133B oligonucleotide arrays (Affymetrix). Further details about the experimental design are presented in the original analysis (Calvano et al., 2005). The transcriptional analysis generated expression measurement data of over 44000 probe sets in total, which is also publicly available through the GEO Omnibus Database^2^ under the Accession No: GSE3284. Integration and analysis of transcriptomic/metabolomic data from different tissues. We presented extensive data analysis and modeling associated with the transcriptional response to endotoxin in humans (Foteinou et al., 2009a,b,c, 2010, 2011; Dong et al., 2010; Nguyen et al., 2011, 2013; Yang et al., 2011b; Scheff et al., 2013).

### Integration and Analysis of Transcriptomic/Metabolomic Data

Data analysis for both transcriptomic and metabolomic datasets started first by filtering for differential expression over time. Transcripts and metabolites with differential temporal profiles were determined using EDGE software (Leek et al., 2006). The significance cut-off for the transcriptomic dataset were *p* < 0.05 at 0.10 false discovery rate. To determine the potential co-regulatory relationships, differentially expressed transcripts and metabolites with differential temporal profiles were hierarchically clustered using clustergram function in the Bioinformatics toolbox of MATLAB (Mathworks, Natick, MA, USA). The two clusters were obtained by using correlation as the distance metric.

Pathway enrichment analysis of genes in the clusters were completed in Enrichr (Chen et al., 2013) using the gene-set libraries of Kyoto Encyclopedia of Genes and Genomes (KEGG) (Kanehisa and Goto, 2000). Three types of enrichment scores are calculated by Enrichr to assess the significance of overlap between the input list and the gene sets in each gene-set library for ranking a term’s relevance to the input list. These are Fisher exact test, *z*-score of the deviation from the expected rank by the Fisher exact test, and a combined score that multiplies the log of the *p*-value computed with the Fisher exact test by the *z*-score. The pathways which have a combined score higher than 1.0 were called significant. The combined score was devised because Fisher exact test had a slight bias that affects the ranking of terms solely based on the length of the gene sets in each gene-set library (Chen et al., 2013).

The goal in the current analysis was to reveal transcriptional regulation of leukocyte metabolic processes, specifically, then to assess if these regulatory patterns might have been affected by concurrent fluctuations of metabolite levels in the surrounding plasma along with the initial stimuli. For this purpose, we opted to focus the transcriptional analysis to the genes that are associated with metabolic processes *only*. Therefore, any differential transcripts which code for genes that are not associated with any of the metabolic pathways were filtered out. Gene set libraries and pathway classifications in KEGG database were used as reference at this filtering process. Then, clustering analysis was repeated for the remaining transcripts. Clustered metabolism-associated genes were functionally annotated through Enrichr similar to the analysis of the complete transcriptome described above. A detailed account of the results is described in length in (Kamisoglu et al., 2013, 2014, 2015a).

The study aimed at defining the impact of altered plasma composition on the transcriptional response of leukocytes during an inflammatory challenge. Earlier transcriptional studies (Calvano et al., 2005) highlighted components of pro- and anti-inflammatory processes, whereas the integrative analysis focused on metabolic processes controlled at the transcriptional level and enabled the development of guiding principles driving the impact of the immediate leukocyte environment.

One of the key observations indicated that leukocytes tune the activity of lipid and protein associated processes at the transcriptional level in accordance with the fluctuations in metabolite compositions of surrounding plasma. A closer look into the transcriptional control of metabolic pathways uncovered alterations in bioenergetics and defenses against oxidative stress closely associated with mitochondrial dysfunction and shifts in energy production observed during inflammatory processes. We observed that in parallel with the peaking lipid and plunging amino acid levels in plasma, lipid associated metabolic pathways were activated while protein translation machinery slowed. We hypothesize that drastic changes in the immediate environment of the leukocytes might have an adaptive effect in this response in conjunction with the initial stimuli. Furthermore, focusing only on metabolism associated transcripts uncovered alterations in bioenergetics and defenses against oxidative stress that can shed light into the mechanisms underlying mitochondrial dysfunction and shifts in energy production observed during inflammatory processes. Besides describing the metabolic response of human body to a basic inflammatory cue at the systemic level together with affected immune mechanisms, this study can inspire future translational studies as the *-omics* analyses becomes more routine in clinical practice. Blood is one of the most rapid and least invasive biological samples collected from patients, yielding useful information about the state of the body. Benchmarking the metabolic state of the system and transcriptional state of the immune cells by a single biological sample may expedite clinical decision making and help reduce mortality in critical cases. The studies of (Langley et al., 2013, 2014), which formed the basis for our comparative analysis between endotoxemia and sepsis (Kamisoglu et al., 2015a), specifically aimed at benchmarking the metabolic state and identifying critical biomarkers. The analysis presented earlier, based on (Kamisoglu et al., 2015a), points to directions where control studies (human endotoxemia) with established links to clinical cases (sepsis) can be used to elaborate on likely markers and/or expected dynamics enabling easier translation of clinical data.

The integration of multiple temporal data of diverse nature, raises several issues: the temporal resolution of sampling is often assumed. The hypothesis is that if one is interested in deciphering the dynamic interactions between an input and its output, the sampling frequency and timing of the sampling need to be similar. However, data acquisition in living systems reflects a balance between what is physiologically appropriate and what is realistically feasible, accounting not only for cost, but for other practical and ethical constraints. In human studies for example, sampling frequency is limited due to ethical and other constraints. In animal studies, practicality of experiments and their cost often limit sampling, especially if/when it involves sacrificing the animal, in addition to ethical considerations evaluated by the appropriate IRB. Therefore, multiple confounding factors exist, including pre-existing conditions, multiple drugs, age, sex, ethnicity etc. It is the hope that large scale studies will provide some stratification and generate somewhat more coherent data. Furthermore, deciphering evolution of dynamics following an external perturbation needs to take into consideration that the “homeostatic” dynamics may in fact demonstrate a baseline dynamics response – most likely in the form of circadian variation (Almon et al., 2008a,b; Nguyen et al., 2010b, 2014b; Ovacik et al., 2010; Rao et al., 2016b). Therefore, deviations from homeostasis in response to a pharmacological agent need to consider the homeostatic dynamics as well. This is evident not only in terms of data, but also in the context of model development and its implications (Scheff et al., 2010b; Mavroudis et al., 2013, 2015; Pierre et al., 2016; Rao et al., 2016a).

## Model-Based Integration of -*Omics* Data

Modeling the responses of the body to a drug is a fundamental process in the drug development and it helps us quantitatively reflect the time-course of the effects of drug on the body. Building and successful utilization of these models allow quantification of drug-system interactions and prediction of both therapeutic and adverse effects (Mager et al., 2003; Felmlee et al., 2012). Diverse physiological effects of synthetic glucocorticoids are the subject of many of these pharmacokinetic and pharmacodynamic modeling efforts. A series of models were developed to explain the dynamics of receptor regulation and enzyme induction following MPL administration (Sun et al., 1998; Ramakrishnan et al., 2002). The models progressively enhanced to capture the effects of the drug under several doses and dosing regimens. However, these models were based on the data generated by traditional message quantification methods that only allow measurements of single end points. Together with the analysis of MPL effects on various tissues via high-throughput technologies such as gene microarrays (Almon et al., 2007a,b), the diversity of the available models increased. The fifth-generation model that described the simple gene induction by MPL was expanded to several pharmacogenomics models that may explain the response of all the hepatic genes with various dynamic patterns (Jin et al., 2003). Earlier modeling analyses have primarily concentrated on the effects observed at the gene expression level (Almon et al., 2003, 2005, 2007a,b; Jin et al., 2003; Nguyen et al., 2010a, 2014a). The next quest in the development of more comprehensive models is the incorporation of information at the protein expression level. This information, made available by a novel high-throughput and reproducible method, allows the temporal profiling of tissue proteome (Nouri-Nigjeh et al., 2014). A future direction we envision is to achieve integration of this information from complementary studies with a model-driven approach. With this, the current PK/PD models of MPL response could be augmented to reflect the physiological response observed at the protein expression level.

As our results demonstrated earlier, transcriptional and proteomic expression patterns roughly correlate for some of the genes, yet for others, the dynamics are more unexpected. One way to work with the existing PK/PD models would be teasing out the protein counterparts of the transcriptional clusters that are described by the observed dynamics and examining the potential mechanisms that could explain the observed protein expression profiles corresponding to the same genes. Another approach is considering the physiologic response as systems response composed of dynamics of individual elements. However, studies focusing on understanding the relationship between global mRNA transcription and protein translation have produced mixed results, often concluding that the transcriptomic and proteomic data is far from being easily described as complementary (Greenbaum et al., 2003; Hegde et al., 2003; Nicholson et al., 2004; Waters et al., 2006; Haider and Pal, 2013). Nevertheless, both data types reflect the dynamics of the cellular response, thus capture critical information reflecting different facets of the response. The key challenge is realizing that although the various *-omics* (genomic, transcriptomic and proteomic in this case) components at some elementary level augment the number of descriptors, the augmentation is not passive, i.e., it is not simply increasing the dimensionality of the space.

We will present a preliminary study applying this second approach. In our study, the driver of the response is the drug-receptor complex in the nucleus; transcripts and proteins, the nodes of the network, are the individual elements with diverse dynamics. The observed phenotype reflects the systems response arising from the dynamics of these individual elements, and these elements include genes and proteins, directly and indirectly, affected by the MPL. A number of target genes to be included in the network depends on the available biological data, literature information about the interaction of the nodes, as well as the desired complexity level. As more nodes are added into the network, the direct and indirect interactions between the elements of the network, as well as the number of parameters to be estimated, increases.

### Building the Response Network

**FIGURE 5:**
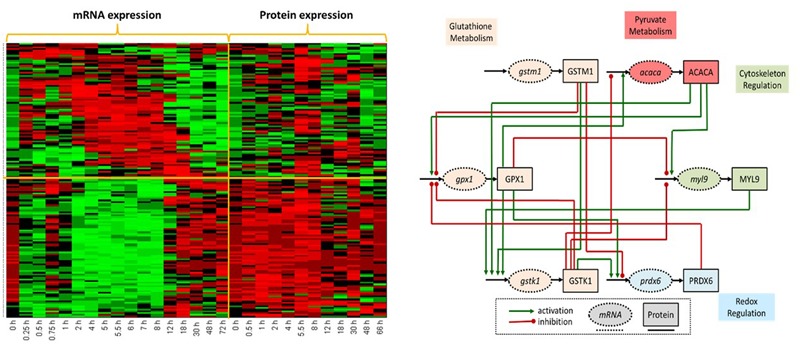
**(Left)** Concurrent analysis of temporal mRNA and protein liver-specific data post MPL dosing revealed two dominant patterns. The first populated with genes whose corresponding mRNA and protein expression profiles were parallel in direction; while in the second directionality was reversed. Heat shock proteins and proteins regulating protein degradation and translation were in the first cluster. The second pattern was dominated by proteins involved in the modulation of oxidative stress, lipid metabolism and bile-acid biosynthesis. Such analyses likely point to transcriptional or post-translational regulatory events. **(Right)** Liver-specific differentially expressed proteins and transcripts following a dose of MP were determined using EDGE and common functionally enriched pathways using Enrichr. Direct and indirect regulatory relations identified using four databases (Biocarta, KEGG, NCI, and Reactome) established a global network subsequently reduced (6 nodes, 16 edges) using a variation of Dijkstra’s algorithm. Network includes: oxidative stress genes in glutathione metabolism and redox regulation critical for the regulation of inflammation and metabolism; core genes in pyruvate metabolism and fatty acid synthesis; regulation of cytoskeleton as well as metabolic genes.

A functional approach was undertaken to construct a *proof-of-concept* initial network. Genes included in this network are selected from the most informative transcripts that are both differentially expressed at the transcriptomic and proteomic levels, **Figure 5** (left). The list was further reduced by focusing on genes functionally related to the major metabolic effects of MPL on metabolism, including hyperglycemia, dyslipidemia and muscle wasting (Schäcke et al., 2002). Genes functioning in glutathione metabolism and redox regulation, associated with oxidative stress, are situated at the major cross-roads critical for the regulation of both inflammation and metabolism (Pearce and Pearce, 2013). These nodes seem to be closely connected to one of the core genes in pyruvate metabolism, also coding for enzymes catalyzing the rate limiting step of fatty acid synthesis. Another critical response to inflammation, regulation of cytoskeleton, although mostly epithelial in origin, is also represented in this network in collaboration with other genes functioning in metabolic pathways.

In order to establish regulatory connections between the sub-set of genes and proteins, we explored several databases, including Ingenuity Pathway Analysis (Calvano et al., 2005), KEGG (Lissauer et al., 2009), Biocarta (Amirian et al., 2011) and PSTIING (Ng et al., 2011), which provide network data. The network was constructed by combining the transcriptomic and proteomic data along with functionally annotated biological information and literature-based functional associations, with emphasis on regulatory relations. Utilizing the regulatory relations between genes/proteins from the databases, first a “global network” in which regulatory links are well known was established. The analysis produced a small, yet complex network (6 nodes, 16 connections) providing an avenue to explore both therapeutic and adverse effects of MPL, **Figure 5** (right). Refinement and further extension of the network through modeling will yield an accurate representation of the complete effects of the drug. Once the sub-set of genes and proteins of interest has been identified, we then need to establish a network structure expressing putative regulatory relations. A variety of computational methodologies for further refining regulatory network structures are discussed elsewhere (Yang et al., 2007b; Foteinou et al., 2009d; Nguyen and Androulakis, 2009; Nguyen et al., 2011; Saharidis et al., 2011).

### Integrating the Network With an Existing PK/PD Model for MPL

Inference (regression) methods (Fujii et al., 2017) correlate inputs and outputs without prior knowledge of the underlying network structure. Instead we pursued a “reaction-based” approach (Newman et al., 2013) which is an *a priori* biological knowledge enabling the quantification of the dynamics (Androulakis et al., 2007, 2013) exploring the principles of mass action (Aldridge et al., 2006) and indirect response modeling (Krzyzanski and Jusko, 1998a,b) for expressing the rates of synthesis and degradation of mRNA and protein. The general structure of the model is composed of three sub-units: (a) the PK of MPL describes a bolus injection which has already been described using a two-compartment model and is maintained throughout (Jin et al., 2003); (b) the PD module describes the transduction cascade leading to the formation of the active, nuclear, complex binding to the GRE (Jin et al., 2003); and (c) the module describing the transcription/translation dynamics. For the latter, we model the transcription/translation process as a dynamic system where the basal rate of transcription of *mRNA* is regulated by the activity of regulating proteins, whereas the translation is proportional to the amount of *mRNA*. The basic formalism 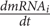 = *k_s,m_ f_i_(P) - k_d,m_ mRNA_i_*; 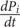 = *k_s,P_ mRNA_i_ - k_d,P_P_i_* reflects the fundamental dynamics of transcription and translation processes through the use of ordinary differential equations (Meister et al., 2013). The function *f(P)* reflects the regulatory action of the various proteins on the transcription *mRNA*. The *f(P)* formally reflects the likelihood of the regulatory events and its functional form will reflect mechanistic interpretations. In its most general form, and based on thermodynamic arguments, it includes regulatory complexes as well as activation or repression of transcription (Bintu et al., 2005a,b):

$$f_{i}(P)=a_{0}(1+DR_{n})+\frac{\sum_{j=1}^{m}\lambda_{ij}\prod_{k\in S_{ij}}P_{k}}{\left(1+\sum_{j=1}^{m}\mu_{ij}\prod_{k\in S_{ij}}P_{k}\right)}.$$

The set *S_ij_* defines a regulatory complex and the coefficients *λ_ij_, μ_ij_* reflect activation and repression constants respectively. Discrete optimization formalisms (Yang et al., 2008b,c; Foteinou et al., 2009d; Saharidis et al., 2011), could also be used in order to augment the network connectivity. In all cases, the basic structure of the network will be as identified via the network analysis, while allowing for the possibility of minor adjustments based on the estimation results. The estimation of the model parameters take appropriate measures of identifiability and uncertainty into consideration in order to achieve robust parameter estimation and is discussed elsewhere (Yang et al., 2011a,b; Wu et al., 2015).

The network structure for **Figure 5** along with the corresponding data of the same figure was used to develop a simple model (Equations 1–9).

(1) $$D=C1\cdot e^{-\lambda_{1}\cdot t}+C2\cdot e^{-\lambda_{2}\cdot t}$$

(2) $$\frac{dR_{m}}{dt}=k_{syn\_Rm}\cdot \left(1-\frac{DR_{n}}{IC_{50\_Rm}+DR_{n}}\right)-k_{dgr\_Rm}\cdot R_{m}$$

(3) $$\frac{dR}{dt}=k_{syn\_R}\cdot R_{m}+R_{f}\cdot k_{re}\cdot DR_{n}-k_{on}\cdot D\cdot {R-k}_{dgr\_R}\cdot R$$

(4) $$\frac{dDR}{dt}=k_{on}\cdot D\cdot {R-k}_{T}\cdot DR;$$

(5) $$\frac{dDR_{n}}{dt}=k_{T}\cdot DR-k_{re}\cdot DR_{n}$$

(6) $$\frac{dmRNA}{dt}=f-k_{dgr\_m}\cdot mRNA$$

(7) $$f=k_{P}\cdot (1+DR_{n}+\sum_{j=1}^{N}a_{ij}\cdot P_{j})$$

(8) $$a_{ij}=\{\begin{array}{c} 1,\;if\;protein\;j\;regulates\;gene\;i\\ 0,\;otherwise\;\;\;\;\;\;\;\;\;\;\;\;\;\;\;\;\;\;\;\;\;\;\;\;\;\;\;\;\;\; \end{array}$$

(9) $$\frac{dP}{dt}=k_{syn\_P}\cdot mRNA-k_{dgr\_P}\cdot P$$

In this model, the PK and PD of MPL, i.e., the equations for *D, R_m_, R, DR* and *DR_n_*, and their corresponding kinetic parameters, are as established earlier in Sukumaran et al. (2011). The kinetics for mRNA follow standard mass action kinetics with a 0th order transcription and 1st order degradation. The transcription, however, is conditionally regulated by MPL and/or other proteins, depending on the network structure expressed through the matrix *a_ij_*. The dynamics of protein synthesis was assumed in its simplest form (1st order translation and degradation). The normalized data associated with the mRNA and protein levels of the network in **Figure 5** were used for estimating the parameters involved in the mRNA and protein dynamics only (the remaining were fixed based on prior deconvolution of the PK/PD model). MPL plasma concentration (*D*) exhibit a biexponential decline. Following the binding of the drug to the glucocorticoid receptor (*DR*), this complex translocates into the nucleus [*DR(N)*] and acts as the driving force for MPL-induced response patterns. Firstly, this effect is observed as inhibition of mRNA expression for the glucocorticoid receptor (*R_m_*), and consecutively the receptor protein (*R*). *DR(N)* is also introduced as a stimulatory factor to all of the nodes in the network. Level of mRNA expression is modeled to be controlled by the presence of drug-receptor complex in the nucleus together with indirect interactions between the proteins of the network. Degradation of mRNA, protein translation from mRNA, and protein degradation are all modeled as linear processes.

**FIGURE 6:**
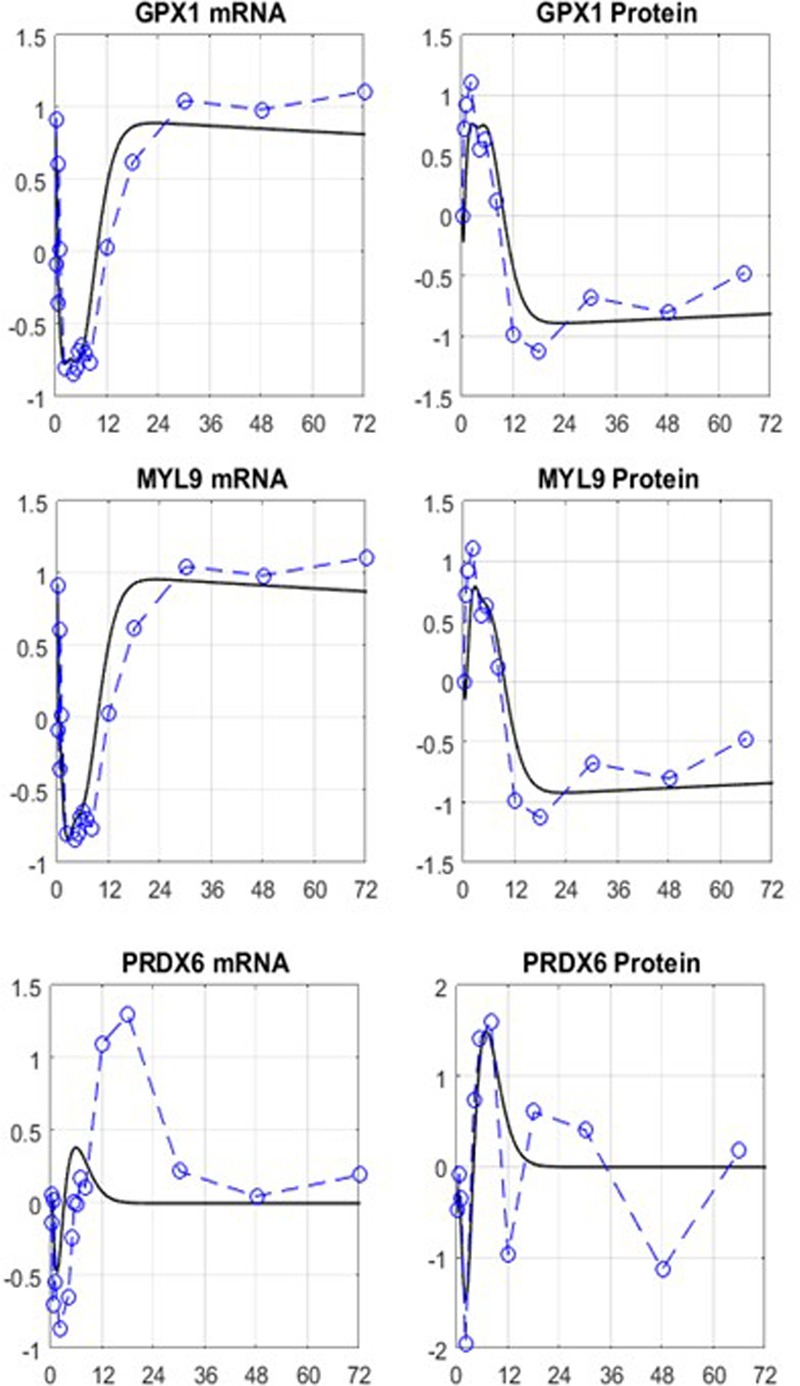
**Preliminary results indicative of the potential to capture the complex dynamics of the interplay between the PK dynamics of MPL and the putative mRNA/protein cross-regulation in the form of networked interactions.** Despite the simplicity of the model equations, key characteristics are captured. The simple form of the added model of regulation clearly needs to be augmented. Furthermore, the preliminary calculations did not take into account the error in measurements. Open symbol/dashed line: data, solid line: model.

The initial, motivating, results, **Figure 6**, are only beginning to scratch the surface and point to directions for improvement, as they should with any iterative model development process. In general, for most network elements early dynamics seem to be represented well compared to later fluctuations in the response. However, significant improvements and refinements are expected. We anticipate the need to develop complex representations, likely requiring precursor and receptor-mediated indirect responses (Sharma et al., 1998; Sharma and Jusko, 1998; Almon et al., 2002; Hazra et al., 2007). It is important to realize that model structure adjustments have the potential to lead testable hypotheses in the laboratory to further elaborate on the actual PK/PD. Given prior experience with both the modeling principles (Wang et al., 2012; Stamatelos et al., 2013; Mavroudis et al., 2014; Mavroudis et al., 2015) as well as the associated parameter estimation of such pharmacology models in terms of structure (Yang et al., 2007a, 2008b,c, 2009a; Foteinou et al., 2009d; Saharidis et al., 2011) and parameter identification (Yang et al., 2011a,b; Wang et al., 2012; Stamatelos et al., 2013; Wu et al., 2015), a reasonable estimation problem can be formulated. Some of these efforts might include introducing time delays to the elements which demonstrate more pronounced dynamics in the later phase of the response. The synthesis of the network mRNAs currently involves linear relationships. Michaelis–Menten kinetics can be introduced to allow self-limiting responses. Finally, the network structure can also be reshaped by eliminating the nodes that are not insightful as well as introducing new nodes that carry important regulatory information about the existing parts of the network. Once the model is matured to fully capture the experimental data, it can be utilized to make predictions about long-term effects, or different delivery kinetics.

Another layer of information that can be incorporated into this model in the future is the metabolome layer. As we have previously pointed out, gene expression signatures give information about the lowest level of organization, shedding light on the origin of a specific phenotype. Proteomics provide information about the abundance of proteins, elucidating the next level up from gene expression data. The integration of these fields provides a unified picture of cellular-level responses from transcription through translation. However, there are multiple other levels of processes that control the sequence of events from the translation of a protein until it becomes a fully functional piece of the organism that can shape processes affecting the metabolic phenotype. Metabolomics complements these more traditional -*omics* techniques by allowing the investigation of properties that cannot be directly assessed through gene and protein expression. Integration of metabolomics with transcriptomics and proteomics can help make the relationship between the levels of information produced by each technique clearer. Changes in gene expression levels and protein concentrations can be linked to physiologic changes and interpreted in the biological context.

Future metabolomic studies in the same animal model can elicit the metabolic shifts occurring in response to MPL administration that ultimately cause the development of the adverse effects. Careful assessment of the connections of these shifts with the defined alterations in hepatic gene and protein expression levels can help identify the critical nodes that control the metabolism-associated adverse effects of the drug. Importantly, the indirect effects of the drug on whole body metabolism through altering the microbiome would have to be considered here as well, since the symbiotic organisms might have tremendous influence on shaping the metabolic response to the drug. Nevertheless, integration of information from the whole-body metabolism with existing information on the hepatic response to MPL can be useful in multiple ways. Firstly, alterations in the critical nodes that are linked to long-term adverse effects can be identified and adjunct therapies that can alleviate these alterations can be devised. Secondly, patient populations which would be more susceptible to experiencing those adverse effects, or who have better drug response, can be pre-determined based on their genomic profiles. Thirdly, more realistic models of drug response can be designed integrating information from this ultimate phenotypic level and be used to evaluate different scenarios, helping in the design and development of better therapies.

## Conclusion

The purpose of this review article is to provide a perspective on the opportunities and challenges associated with the integration of disparate *-omics* data sources. Without a doubt the next frontier in PK/PD modeling will take advantage of our increasing abilities to incorporate biological information across multiple and diverse layers of physiologic organization. Although the richness of the data is impressive, rationalizing the content in a systematic and coherent manner remains a challenge. Given the overall difficulty of the problem, upgrading the information content of the data is a major challenge.

In this review, we have attempted to discuss three topics, based on our own experience. We discussed challenges associated with integrating *-omics* information within and between tissues. Interestingly, the nature of the data, to some extent, drives the type of question and the corresponding methods needed for answering the question. Finally, we provided a brief summary of what we feel is a promising new frontier: integrating *-omics* information in a model-based manner. The expectations are twofold: (1) we may achieve a better rationalization of the information; and (2) perhaps more importantly, we may be able to further advance the frontiers of PK/PD modeling which could have significant impact. This preliminary work introduces an approach for bridging the classic PK/PD modeling efforts with the multi-level systems response. This allows us to explore the paths of utilizing the vast amount of information made available by new *-omic* profiling tools. These tools make it possible to evaluate the response as a whole at a certain biological level over time. The model-based integration approach discussed here ultimately aims to connect this valuable information coming from multiple layers in a useful framework which reflects the continuity of biological events in response to pharmacological stimuli. Achieving such integration will allow the development of model-based approaches for rationalizing the genomic, transcriptomic and proteomic data in the context of integrated dynamic regulatory network models, critical for the development of MPL PK/PD/pharmacogenomics models, enabling us to move beyond using *-omics* as a complex descriptor toward the development of pharmacologically relevant and predictive computational models.

Without a doubt, we are only scratching the surface. Numerous challenges and open questions remain, charting an exciting future. To name a few, our case studies present a modeler’s idealized scenario: relatively homogenous cohorts, whether a single animal strain in our MPL studies or a population of relatively healthy humans in our human endotoxemia studies; single dosing with a single pharmaceutical agent. Hidden within is reasonable biological variability; however the main focus of these designs was to tease the agent’s primary effects. As we consider moving integrative *-omics* to the next level, we must expect a number of challenges: (1) as we hinted in the comparison of metabolomics profiles between a controlled human endotoxemia study and the clinical cases, *-omics* data from clinical studies and/or patient population will, unavoidably, express and capture many confounding factors beyond responses elicited by the agent under study; (2) circulating, i.e., systemic, markers pose, as discussed in the manuscript, additional challenges since the tissue-specificity is lost, complicating further the interpretation of the observed responses in a cause-and-effect sense; (3) the temporal granularity of the data will remain a key challenge. The disparities in temporal resolution of the responses at different physiologic levels will further complicate any data driven-approach. In such cases, it is likely that methods aiming at features and/or models – as discussed in the paper – will prove more beneficial; (4) patient history, including medication, will constantly nuance the data obtained; (5) despite the ability to probe an ever increasing number of likely biological descriptors and mediators (genome, transcriptome, proteome, metabolome, epigenome, fluxome, etc.) leading to an increase in the dimensionality of the “input” space, the actual “output” space, that is the number of subjects, volunteers and/or patients, being sampled will always lack, especially if appropriate population stratifications are implemented. This is a classic problem in machine learning often referred to as classification/feature selection in “almost empty spaces” (Duin, 2000) Therefore, key challenges will remain, primarily focusing on the many aspects of the heterogeneous nature of the data. However, as our ability to collect, archive, annotate and query new challenges and opportunities emerges (Toga et al., 2015; Dinov et al., 2016), large cohort studies are already emerging (Hood and Price, 2015).

Finally, one of the key themes of this presentation is that integration can occur at two levels: (a) the level of the features of the data, or (b) the level of the features of the models that could describe the dynamics of the data. Each method offers distinct advantages and challenges. Considering features of the data, characteristics of the responses and likely important biomarkers are able to describe intricacies of the response. Features of the models underlying the dynamics of the data, on the other hand, enable a likely quantification of cause-and-effect relations, as well as the likelihood of expressing, and predicting, complex dynamics and emergent behaviors, not necessarily obvious while studying the features themselves. However, it is important to realize that the approaches are complimentary and are often combined to improve the overall effectiveness of the analysis. To some extent, effectiveness is also an emergent property of the concurrent and multi-prong analysis of information-rich *-omics* data.

## Author Contributions

KK, AA, and TN: performed calculations, conducted analyses and edited the manuscript. RA, SC, SC, DD, and WJ: edited the manuscript. IA: conceived the studies, and developed the manuscript.

## Conflict of Interest Statement

The authors declare that the research was conducted in the absence of any commercial or financial relationships that could be construed as a potential conflict of interest.

## Footnotes

1 www.ingenuity.com

2 http://www.ncbi.nlm.nih.gov/geo/
